# Trace Elements Anomalous Concentrations in Building Materials—The Impact of Secondary Mineralisation Processes

**DOI:** 10.3390/ma17163909

**Published:** 2024-08-07

**Authors:** Agnieszka Pękala, Piotr Koszelnik, Michał Musiał, Tomasz Galek

**Affiliations:** 1 The Faculty of Civil and Environmental Engineering and Architecture, Rzeszow University of Technology, Powstancow Warszawy 6, 35-959 Rzeszow, Poland; pkoszel@prz.edu.pl (P.K.); mmusial@prz.edu.pl (M.M.); 2 The Faculty of Mechanics and Technology, Rzeszow University of Technology, Kwiatkowskiego 4, 37-450 Stalowa Wola, Poland; t.galek@prz.edu.pl

**Keywords:** raw materials, environmental monitoring, cadmium, arsenic, micropollutants, lignite

## Abstract

The Pb, Cr, Cd, Ni, Zn, Cu, Co, As, Sr, Ba, and Zr content has been determined in the tested rock raw materials. The concentration of cadmium (Cd) was found to be elevated in all types of rock materials and was found on average to be: 1.39 mg/kg in limestones, 0.86 mg/kg—sandstones, 0.44 mg/kg—diatomites, 0.55 mg/kg—opoka rocks, 0.89 mg/kg—marls, 0.21 mg/kg—gaizes 0.42 mg/kg—kaolin clays, and 2.13 mg/kg—decalcified opoka rocks. Higher concentrations of arsenic (As) have also been recorded in sandstones and diatomites, as well as lead (Pb) in limestones and sandstones. The results obtained indicate that the anomalous level of elements is of natural origin and the results of identified secondary mineralisation processes that have affected the tested materials. Pyritization and sulfatization processes have been detected. Mineralogical research has shown that these processes can be associated with the activity of biochemical processes caused by the decomposition of the soft tissues of animal organisms and the organic substances of plant origin that fill the stylolites. It has been shown that the content of strontium (Sr) increases in geologically older Jurassic formations compared to younger Cretaceous formations, which can be used in the monitoring of building materials.

## 1. Introduction

The global construction product control system pays particular attention not only to the broadly understood safety of residential buildings and other construction facilities, but also to the health, durability, energy saving, environmental protection, and other requirements of the common market that are important from the point of view of social and individual interest. Established legal provisions influence the requirements for materials themselves and the elimination of construction products that contain excessive amounts of harmful ingredients, even if they are of natural origin [1]. Many countries are working to establish an environmental assessment system for materials used in construction. In these works, attention is paid to the determination of chemical elements of recycled construction aggregates and waste mineral raw materials used in construction [2,3]. A system for evaluating the materials used in construction must take into account their impact on the natural environment. This assessment requires a detailed analysis of the internal structure of the material and the external factors that influence the possible release of hazardous substances. Not only must composites based on harmful materials be tested, but so should materials of natural origin. Material analysis must include an assessment of the material matrix in terms of chemical, physical, and mechanical properties. This is important not only in terms of industry but also, above all, ecology. The influence of external factors, in particular, the water environment (precipitation, aggressive groundwater, and other aggressive fluids), may release additional amounts of components originally related to the structure of the material [4,5]. The necessary basic research includes the assessment of the content of trace elements in mineral raw materials in terms of environmental protection, especially when they can be used as construction and technological materials. Some trace elements enter the structures of minerals, are relatively resistant to changing environmental conditions, and do not pose a threat to the biosphere. The remaining part occurs in the form of carbonates, phosphates, sulphides, oxides, etc., and is also adsorbed by aluminium minerals, organic matter, as well as hydrated iron and manganese oxides [6,7,8]. Before using rocks in residential and commercial building materials, from the point of view of radiological protection, it is necessary to monitor natural radiological nuclides [9,10,11,12]. Radiological hazard assessment is also carried out in the case of the use of minerals, for example, mica as a raw material in the cement and concrete industry [13].

The subjects of the study are sedimentary rocks originating from the Mesozoic basement located in the Mesozoic-Neogene contact zone in the Bełchatów lignite deposit (Central Poland). The sediments located there are characterised by diversity in terms of geological forms of occurrence, with differences also in the age and type of rocks that form the basement and consequently in the diversity of weathered formations located in the contact zone of the Neogene and Mesozoic basement rocks.

The use and management of lignite series bedrocks in open-pit lignite mining is very important in many economic aspects. Natural resources must be protected by limiting the area of mining areas. It is important both economically and for environmental protection to use some of the obtained raw materials for the production of building and pro-ecological and pro-ecological materials. Previous works have presented the petrographic nature of these rocks, possible directions for their use, and their lithological diversity [14,15,16]. Research and the current situation show that it is possible to use some of the rocks examined from the Mesozoic-Neogene contact zone from the Bełchatów lignite deposit in the economy. Due to the intensive development of building materials, the cement and lime industries, and the increasing demand for carbonate sorbents, limestones present great opportunities in this direction. The opoka rocks and marls also identified in this region showed the possibility of being used in ecological technologies as mineral sorbents; in the ceramic industry as a component for the production of glazed cladding tiles, coloured floor tiles, porcelain and sanitary porcelain, as well as various stoneware products; or in the direction of construction materials [16]. Geochemical research on these rocks is a very important and necessary aspect of environmental protection. Rock raw materials extracted from deposits are often collected in landfills and heaps, where the concentration of elements may be many times higher than that under natural conditions. From the studies carried out to date that present the geochemical nature of these rocks, the content of thorium and uranium was determined. Analyses of the content of radioactive elements were carried out for 50 samples of Jurassic limestone, 18 samples of kaolinite clays, and 24 samples of silica raw materials, represented by opoka rocks, diatomites, gaizes, clastic rocks, and 6 samples of marl. The highest concentrations of these elements were found in clay raw materials. Their value ranged from 8 to 12 mg/kg for thorium and from 2.3 to 3.5 mg/kg for uranium, respectively. In carbonate formations, the thorium content ranged from 0.5 to 2.1 mg/kg, and the uranium content ranged from 0.5 to 2.2 mg/kg. Of the group of silica raw materials, the highest concentrations were in diatomites, where the thorium content ranged from 1.5 to 1.8 mg/kg and uranium ranged from 1.3 to 1.7 mg/kg. The concentrations of radioactive elements found in the tested mineral deposits did not exceed the permissible values. The only thing worth noting is the relatively high concentration of thorium and uranium in the opoka rocks. This situation can be explained by the presence of minerals such as monazite in these rocks [17]. It was also shown that the dominant element in transitional formations is strontium, which amounted to 295 mg/kg in the opoka rock and 362 mg/kg in the decalcified opoka rock [18]. However, detailed geochemical analyses of other lithological types of rocks are lacking. The present paper aims to fill the gap in this area.

## 2. Research Methodology

The research methodology used included the following:

- Determination of the chemical composition was carried out by the atomic absorption spectroscopy (ASA) method using the PHILIPS PU 9100Xi Camera SX-100 spectrophotometer and the ICP.ULTIMA 2 HORIBA JOBIN-YVON sequential plasma spectrometer with the possibility of retrospective analysis, operating in the spectra range from 160 to 800 nm with the possibility of expanding them at any time to the range of 120–800 nm. The software compatible with the ICP spectrometer enables recording of the full spectrum in less than 200 s at the full resolution of the spectrometer. The tests were carried out at the accredited Aerospace Materials Testing Laboratory of the Rzeszów University of Technology.
- Scanning microscopy was carried out using a MIRA3 Tescan electron microscope (SEM). To determine the chemical composition, field emission and an X-ray detector (EDS) from Oxford Instruments were used. The research preparation required sputtering the samples with a layer of gold with a thickness of approximately 30–45 nm. This process was carried out on a vacuum sprayer. Sample imaging was performed at four magnifications of 2 k, 5 k, 20 k, and 50 k times. The electron acceleration voltage was selected in the range of 10 to 20 kV. Elemental mapping was performed at 1000× magnification. The average time for one mapping is approximately 10 min. X-ray detection covered the energy range from 0 to 10 keV. The surface distribution of the elements was made at an image resolution of 1024 × 1024 pixels. The time to count the signal to the spectrum from one pixel was 500 microseconds. The elemental composition was the average value of the entire map.
- An X-ray spectrometer (EDX Genesis) and backscattered electrons (BSE) detector were used for point analysis of mineral phases and obtaining microphotographs illustrating the phase differentiation of solid surfaces.
- Microscopic observations in polarised transmitted light were carried out using microscopes—Panthera TEC POL Trino equipped with a high-sensitivity Pro-S5 microscope camera with an sCMOS matrix and a Global Shutter shutter type.
- Observations and photographs of microsection were obtained using an Olympus SZX7 stereoscopic microscope, equipped with a Galilean optical system with plan apochromatic lenses, free from distortion, along with a microscope camera and software enabling image acquisition and measurements.

## 3. Research Stages

### 3.1. Fieldwork

**Figure 1:**
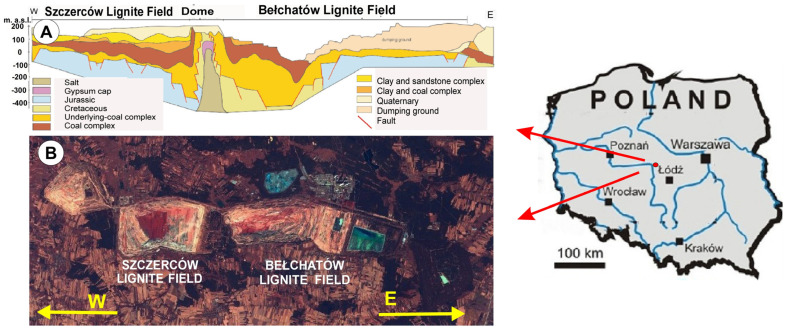
Area of research carried out. Bełchatów lignite deposit (**A**) cross section through the Bełchatów lignite deposit [21]; (**B**) location of the study area depicted in the colour composition of a Sentinel satellite image.

The Bełchatów lignite deposit is located in central Poland (Central Europe), in the Polish Lowlands, in the Szczecinsko-Łódzko-Miechowskie Synclinorium. It has the character of a tectonic deposit. The tectonic trench in which the studied deposits were formed is limited by tectonic lines approximately 35 km long and 1 to 2 km wide. The average thickness of the zone is 150 to 200 m, reaching a maximum of 390 m. Its range in the western part of the country is 72 km (Figure 1A) [19]. There are three areas in the Bełchatów lignite deposit: the Szczerców Field, the Bełchatów Field and in the east, the Kamieńsk Field (Figure 1B), from which, apart from the main mineral, accompanying minerals are also extracted. Annually, 38.5 million tons of lignite and 100–120 million m^3^ of rock removed from the overburden are extracted from the Bełchatów deposit [20]. The open-pit mining nature of the deposit means that the open pit of the Bełchatów field is approximately 300 m deep and the heaps are 170 m high, from which over 50% of the raw material extraction comes. The advancement of exploitation means that rocks exposed at the contact of Neogene formations with the Mesozoic substrate are becoming increasingly popular.

During field work and while drilling the drill cores, approximately 100 samples were taken for testing. Research material came directly from the Bełchatów open pit and from drill cores that preceded the mining front.

**Figure 2:**
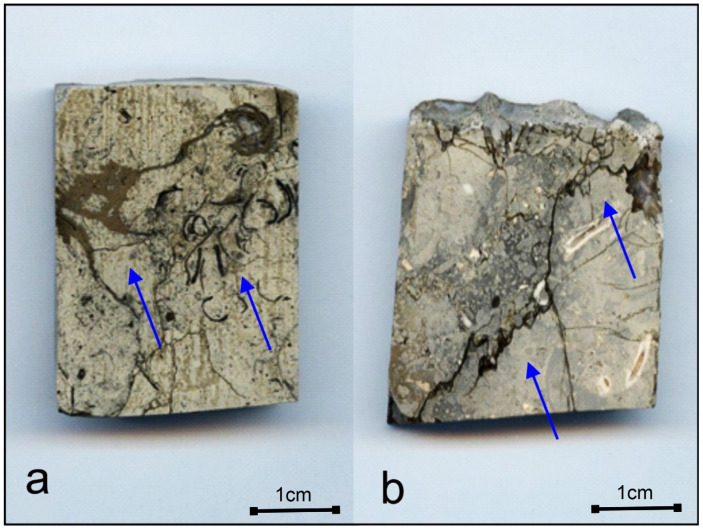
Limestone microsections. Iron sulphides filling of oncoids (**a**) stylolite seams (**b**), (blue arrows).

The material collected shows lithological and stratigraphic diversity. The oldest formations are represented by Upper Jurassic limestones. They occur in the form of thick layers, several metres thick. They represent the Mesozoic substratum. They are very broken. Only locally can you see places where these rocks become compact and hard. These are mainly detrital limestones, cream, beige, or grey in colour. This colour variability was best visible on the limestones exposed directly in the “Bełchatów” outcrop. They become darker towards the bottom. This is the effect of near-surface weathering. The detrital material in the limestones is represented by numerous marl intraclasts, crinoids, bivalves, and oncoids. Some of them show pseudorubble and wavy structures of compaction origin. Oncoids are uneven, pinnate, and have numerous stylolite seams and cracks filled with iron sulphides (Figure 2a,b).

Microfaunal studies in the limestones, indicating the age of the Upper Jurassic (Kimeridgian), revealed skeletal elements of echinoderms and foraminifera: *Lenticulina muensteri*, *Lenticulina tricarinella*, *Lenticulina prima*, *Lenticulina* sp. *Tracholina solecenisis*, *Paleogaudryina varsoviensis*. The marls and marly opoka rocks were found in the profiles in contact with the limestones. Spotted or argillaceous marls of dark grey also formed thick-bedded inserts between the limestones. These formations were characterised by numerous interbed cracks and frequent vertical cracks. The marls contained admixtures of sandy fraction with glauconite, occurring in the form of laminates and streaks. In the cement in marly deposits, in the fracture zones, cracks filled with coarse crystalline calcite and ceslestine were recorded. Some marls showed features of silicified marls. This mineralisation process was also observed in the case of the opoka rocks, a significant increase in the hardness of the rocks and difficulties in mining and extracting them were observed. The Maastrichtian opoka rocks are light grey and white in colour with numerous small fragments of inoceram. These formations that occur in the upper part of tectonic megabreccia zones showed strongly decalcified features. The opoka rocks are enriched with fine plant detritus and sandy material. There are visible penetration structures and feeding channels in the fragments. Micropaleontological analyses revealed a set of macro-remains indicating a Maastrichtian-Campanian age: In addition to foraminifera, there are ostracods and sponge needles. Marked: *Gaudryina rugosa d Orb*, *Plectina levis*, *Spiroplectammina baudoniana*, *Arenobulimina presli*, *Gyroidinoides globosus.* The foraminiferal community was dominated by small forms: *Tritaxia triccarinata Reuss*, *Gavelinella pertusa*, *Gavelinella monterelensis*, *Bolivinoides decoratus.*

### 3.2. Geochemical Research

The next stage included the determination of the following elements: Pb, Cr, Cd, Ni, Zn, Cu, Co, As, Sr, Ba, Zr in: carbonate rocks; limestones; transitional rocks: marls, opoka rocks, and gaizes; clay rocks: clays, clastic rocks: sandstones; siliceous rocks: diatomites; rocks formed in weathering processes: decalcified opoka rocks (Table 1).

#### Geochemical Analysis of the Determined Elements

**Figure 3:**
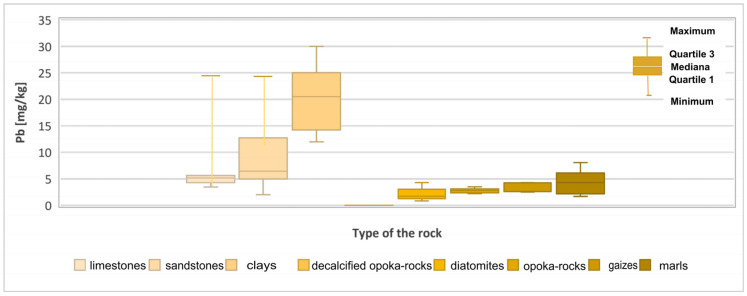
Lead (Pb) content in rock raw materials.

Lead (Pb) in the Earth’s crust occurs almost exclusively as Pb^2+^, occasionally Pb^0^ and Pb^4+^. This element has lithophilic properties. During weathering, usually after oxidation processes in the presence of microorganisms, it is released from sulphur compounds. In sedimentary rocks, the distribution of Pb is related to the presence of primary detrital material. It is easily absorbed by clay minerals, iron oxides and hydroxides, and organic substances. Its average content in the Earth’s crust is 12.5 mg/kg, and it is more concentrated mainly in formations containing minerals such as feldspar and mica. The range of occurrence of lead is highest in clay formations. The highest concentrations (10–40 mg/kg) are in black shales, reflecting the affinity of Pb for organic compounds. Its content in clastic rocks is 5–10 mg/kg, and in carbonate rocks it is in the range of 3–10 mg/kg [22,23,24]. In the rocks examined. The highest average lead content was observed in the kaolin clays and the lowest in the light opoka rocks (Figure 3). The range of occurrence of lead in kaolin clays was 12 to 30 mg/kg and did not exceed the limit values for this element allowed in clay rocks (Table 1). The average lead content in limestones is 7.01 mg/kg and in sandstones 9.42 mg/kg. Values exceeding the level of 20 mg/kg were found in those limestone and sandstone samples where the mineralogical analysis showed the process of mineralisation of sulphides.

**Figure 4:**
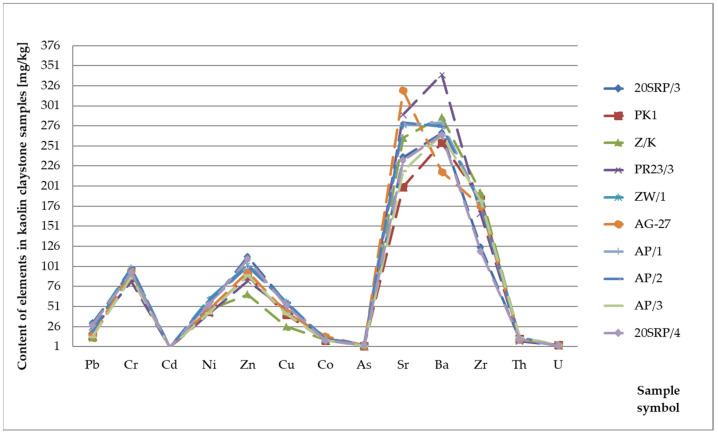
Concentration of trace elements in clays.

Chromium (Cr) is abundant in the Earth’s crust. In sedimentary rocks, Cr may be present, among others, in mineral phases such as chromite, magnetite, and ilmenite. It accumulates mainly in clay rocks (60–120 mg/kg) and decreases in sandstones (20–40 mg/kg) and limestones (5–16 mg/kg). Most chromium minerals are resistant to weathering and, therefore, accumulate in the residue. Under oxidative conditions, the Cr^6+^ cation is formed, which is mobile but is also sorbed by clay minerals and Fe and Al hydroxides [22,23,24]. Chromium has varying toxicity depending on its valence and speciation in the environment. Soluble Cr^3+^ is considered relatively harmless, while Cr^6+^ is highly toxic, causing damage to the liver and kidneys, and is carcinogenic [25]. In the rocks tested, the highest chromium values were recorded in the kaolin clays and were an average of 93 mg/kg (Figure 4, Table 1). All chromium values obtained in individual rock groups did not exceed the permissible values.

**Figure 5:**
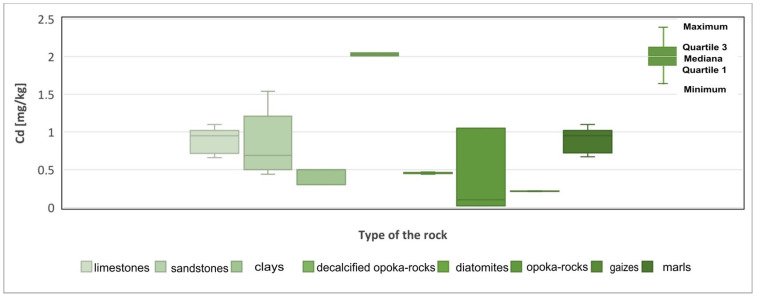
Cadmium (Cd) content in the rock raw materials studied.

Cadmium (Cd) is an element that is highly dispersed in rocks. During weathering processes, it is easily activated and then bound by clay minerals, iron hydroxides, and organic substances. Its content in sedimentary rocks is 0.3 mg/kg in clay formations, 0.05 mg/kg in clastic rocks and 0.035 mg/kg in chemical and organochemical rocks [22,23,24]. In the rocks examined, the concentration of cadmium in individual rock groups is on average: 1.39 mg/kg in limestones, 0.86 mg/kg in sandstones, 0.44 mg/kg in diatomites, 0.5 mg/kg in opoka rocks, 0, 89 mg/kg in marls, 0.21 mg/kg in gaizes, 1.39 mg/kg in clays of kaolin and 2.13 mg/kg in light opoka rocks (Figure 5, Table 1). The results presented clearly indicate that they are overestimated compared to those considered limit or characteristic of the sedimentary rocks.

Nickel (Ni) occurs in sedimentary formations in amounts of 5–90 mg/kg. It shows a decrease in clay rocks (40–90 mg/kg) to sandstones (5–20 mg/kg) and carbonate rocks (7–20 mg/kg). During weathering processes, it is easily activated and in the form of the Ni^2+^ cation, it can migrate with solution over long distances. However, it is often rapidly bound by Fe and Mn hydroxides [22,23,24]. The highest concentration of nickel was found in sedimentary rocks in kaolin clays. It amounted to an average of 50.2 mg/kg (Figure 4). However, this element was not found in the light opoka rocks tested (Table 1). Moreover, none of the tested samples exceeded the permitted nickel content.

Zinc (Zn) has the lowest range in sandstones (15–30 mg/kg) and carbonate formations (10–25 mg/kg). In clay rocks, it is 80–120 mg/kg. The zinc content in the Earth’s crust is estimated at 40 to 80 mg/kg. In weathering processes, all zinc compounds are easily soluble, especially in acidic environments, and the released ions form mineral or organic mineral connections with high mobility. It is rapidly precipitated mainly in the presence of sulphfide ions [22,23,24]. The highest concentration of zinc is found in the tested kaolin clay samples. It ranges from 66 mg/kg to 113 mg/kg and averages 95 mg/kg (Figure 4). The lowest concentrations of this element were found in the light opoka rocks. It ranges from 1.20 to 1.58 mg/kg and averages 1.33 mg/kg (Table 1). Moreover, the permissible content of this element was not exceeded in any of the rock types tested.

Copper (Cu) has chalcophilic properties and a tendency to substitute other divalent cations in minerals and sorption complexes. In weathering processes, all copper compounds are generally easily dissolved, especially in acidic environments. The released copper ions form bonds with anions or organic substances that migrate with solutions. The share of copper in clay rocks is 40–60 mg/kg. In clastic formations, its content ranges from 5 to 30 mg/kg. It reaches the lowest values in carbonate rocks: 2–10 mg/kg [22,23,24]. The average copper content in individual rock lithotypes is 47.6 mg/kg in kaolin clays, 17.21 mg/kg in sandstones, 4.53 mg/kg in opoka rocks, 6.6 mg/kg in limestones, 5.76 mg/kg in marls, 6.74 mg/kg in diatomites, 10.29 mg/kg in gaizes, and 2.35 mg/kg in decalcified opoka rocks. Additionally, variable copper content was observed in the sandstone samples. It ranges from 92 to 1 mg/kg (Table 1). The permissible content of this element was not exceeded.

Cobalt (Co) in sedimentary formations accumulates mainly in clay rocks (10–20 mg/kg). Its content decreases in the sandstones (0.3–10 mg/kg) and limestones (0.1–3 mg/kg). Cobalt is easily mobile in acidic oxidative environments but is not subject to large water migration because it is bound by iron and manganese hydroxides and clay minerals [22,23,24]. The highest concentration of cobalt ranges from 9 to 14 mg/kg in the case of kaolin clay and averages 10.4 mg/kg (Table 1). The determined concentration of this element showed higher values in the case of carbonate rocks. In limestones, it ranges from 0.1 to 6 mg/kg with an average value of 2.76 mg/kg. In marls, cobalt ranges from 0 to 6 mg/kg and averages 2.92 mg/kg. The permissible content of this element was not exceeded.

**Figure 6:**
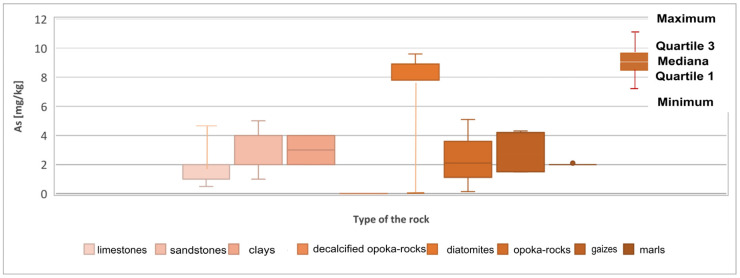
Arsenic (As) content in the rock raw materials studied.

**Figure 7:**
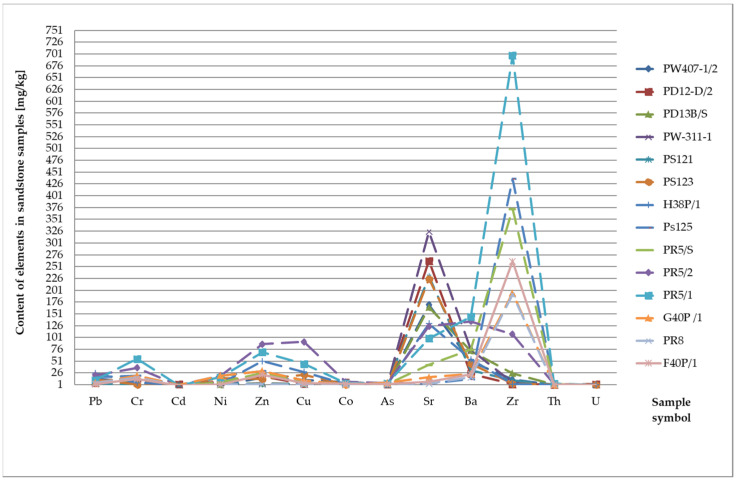
Concentration of trace elements in sandstones.

Arsenic (As) in almost all rocks ranges from 0.5 to 2.5 mg/kg. It is concentrated only in clay formations, most often up to 13 mg/kg [26]. In clastic rocks, its value ranges from 1 to 1.2 mg/kg. In carbonate formations, it is slightly higher, reaching 2.4 mg/kg. All arsenic compounds and minerals are readily soluble. However, its migration is limited by strong sorption by clay minerals, iron and aluminium hydroxides, and organic substances. Moreover, some minerals, for example mica or goethite, have a particular tendency to bind to arsenic and influence its distribution in sedimentary formations [22,23,24]. Due to its harmful impact on human health, arsenic research is also carried out in building materials [27]. Arsenic concentration reaches the highest values in the tested diatomites. It ranges from 0.05 mg/kg to 9.6 mg/kg and averages 6.83 mg/kg (Table 1). Moreover, increased arsenic content was found in the sandstones. The maximum value of this group of rocks is 5 mg/kg with an average concentration of 2.67 mg/kg (Figure 6 and Figure 7). The remaining sediments do not show any exceeded levels of this element.

Strontium (Sr) is a common element. The range of its content in sedimentary rocks is 20–600 mg/kg. During weathering processes, strontium enters the solution, generally in the form of bicarbonate. It is easily sorbed by clay minerals and organisms that create limestone skeletons. Therefore, its content increases in claystones (300–450 mg/kg) and carbonate formations (450–600 mg/kg). In sandstones, it is 20–140 mg/kg [22,23,24]. Strontium was found in all samples analysed (Table 1). The highest concentration of approximately 400 mg/kg was recorded in the decalcified opoka rocks and the opoka rocks. The average value of strontium in these rocks was 359.34 mg/kg in the decalcified opoka rocks and 313.4 mg/kg in the opoka rocks. In marls, it had a value of 308–203 mg/kg, with an average value of 260 mg/kg, and in limestones, 24–375 mg/kg, with an average value of 171 mg/kg. In clay rocks, strontium ranged from 200 to 320 mg/kg, with an average value of 259.6 mg/kg. The average content of this element in the gaizes was 131.6 mg/kg. The lowest values of strontium were found in diatomites—0.05 mg/kg. No supraclark content of this element was found in any of the rock samples tested.

Barium (Ba) in sedimentary rocks is dispersed in the range of 50–800 mg/kg. In clay sediments, its content is 500–800 mg/kg, in clastic formations from 100 to 320 mg/kg and in carbonate rocks from 50 to 200 mg/kg. As a result of its high geochemical affinity for potassium, it occurs in larger amounts in feldspar and mica. It is easily activated in weathering processes and is quickly precipitated in the form of sulphates and carbonates. It is also strongly bound by clay minerals, iron-manganese and phosphate concretions, and sulphur compounds [22,23,24]. Barium was found in all rock lithotypes examined. The highest average values of 273.1 mg/kg will be achieved in clay rocks. Its average content in other types of rocks was: 73.94 mg/kg—limestones, 56.14 mg/kg—sandstones, 38.66 mg/kg—decalcified opoka rocks, 131.1 mg/kg—opoka rocks, 191 mg/kg—diatomites; 60 mg/kg—marls and 144 mg/kg in gaizes. The barium concentration in the rocks samples tested does not exceed the limit values (Table 1).

**Figure 8:**
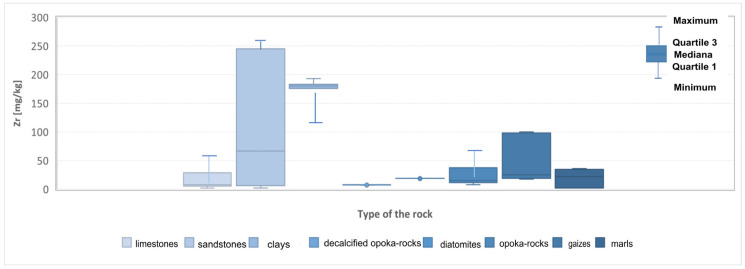
Zirconium (Zr) content in the rock raw materials studied.

Zirconium (Zr) is only partially released during weathering processes. The zircon content ranges from 20 mg/kg in carbonate rocks to 200 mg/kg in clay formations and 220 mg/kg in sandstones [22,23,24]. The zircon concentration reaches the highest values in the tested sandstone samples (Figure 7). Its content ranges from 2 mg/kg to 688 mg/kg, with an average value of 166 mg/kg. Moreover, increased zircon content was found in the limestones and marls. The maximum values in the limestones reached 78 mg/kg with an average contribution of 20.63 mg/kg. The highest zircon concentration in marls is 36 mg/kg and averages 19.2 mg/kg (Table 1, Figure 8).

### 3.3. Mineralogical Research

The conducted mineralogical research allowed us to confirm that the examined rocks were subject to secondary sulfatization and pyritization processes. These processes covered all distinguished petrographic varieties of rocks from the Tertiary-Mesozoic contact zone in the “Bełchatów” deposit. The sulfatization process in the raw materials tested was identified in the carbonate rocks. The occurrence of sulphate residues, gypsum, barite, and celestine, was found within some carbonate bioclasts, in the limestone, marl, and opoka rocks (Table 2).

**Figure 9:**
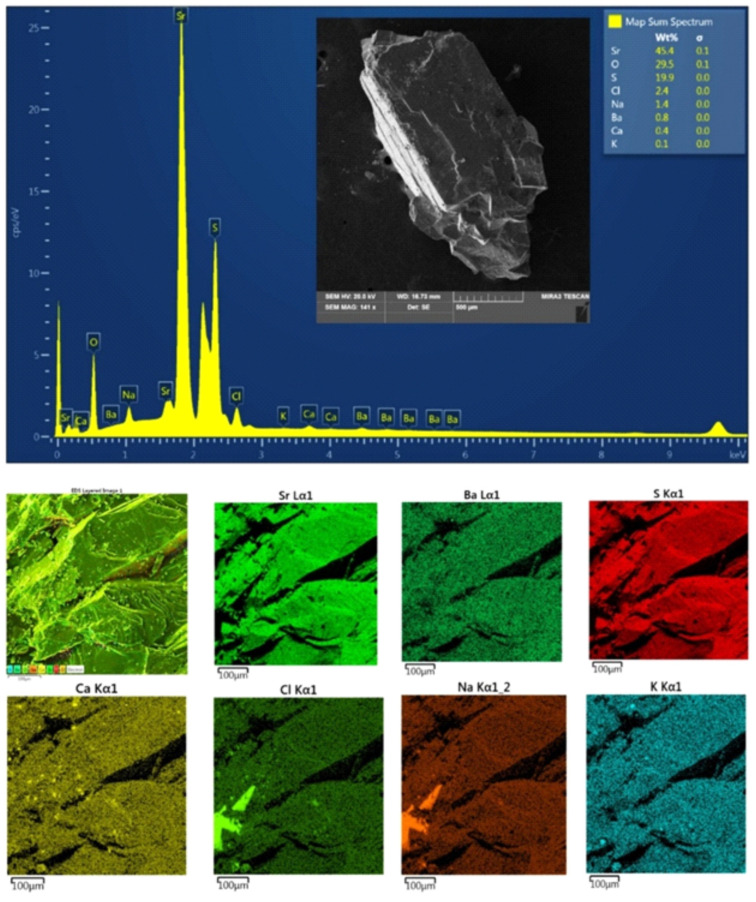
Celestine crystal isolated from a crack in the outcrop. SEM/EDS research.

**Figure 10:**
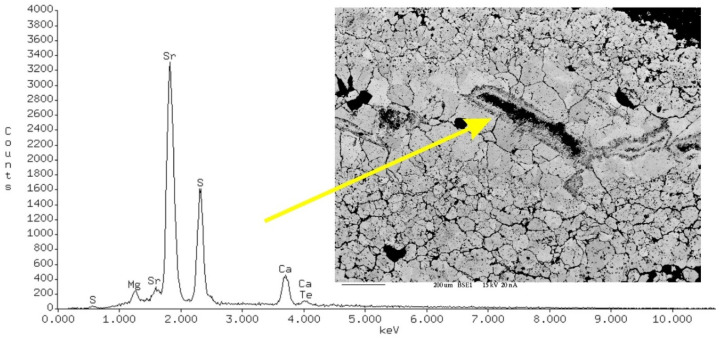
Spectrum of celestine from limestone. EDS/BSE research.

The separated crystals from the voids and crevices were represented by celestine (Figure 9). Research using an X-ray microprobe confirms that these types of mineral phases can be associated with the activity of biochemical processes caused by the decomposition of the soft tissues of animal organisms (Figure 10) and the organic substances of plant origin that fill the stylolites. The decomposition of the soft tissues took place under aerobic conditions with the participation of heterotrophic bacteria. As a result of this process, gas bubbles were released. The metabolic products released, hydrogen sulphide (H_2_S) and carbon dioxide (CO_2_), caused a local increase in acidity. The oxidation of elemental sulphur and hydrogen sulphide, originating from the decomposition of soft tissues by aerobic bacteria, led to a local increase in the concentration of sulphate ions. As a result of the decrease in acidity and dissolution of bioclasts, the concentration of calcium and magnesium cations and bicarbonate ions increased. This process continued until it was saturated with calcium sulphate. As a result, the original metastable calcium carbonate was replaced by sulphates. Biogenic crystallisation of sulphates under aerobic conditions was confirmed in [28]. Strontium is an element that is relatively easily sorbed by organisms that create carbonate skeletons. Celestine has been found in typical marine vertebrates and in the structure of fossil bones [29]. The celestine found in the carbonate remains may have different formation conditions than barite. Barite is the last phase that indicates a radical change in the chemistry of pore waters, from supersaturation in relation to carbonates to supersaturation in relation to sulphates. The next generation of minerals is pyrite, which undergoes oxidation. The bacterial reduction in sulphates led to the crystallisation of pyrite and granular sparite and ended with the beginning of the crystallisation of block sparite [30,31,32].

**Figure 11:**
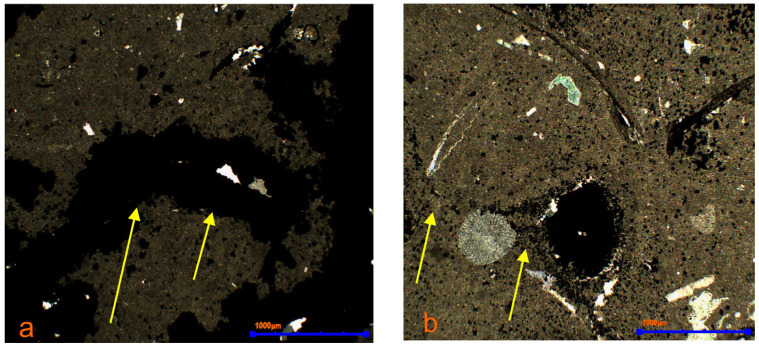
Iron sulphides filling the voids (**a**) stylolite seams (**b**) (markings: yellow arrows). Polarising microscope, 2P. (explanations: P-polariser).

**Figure 12:**
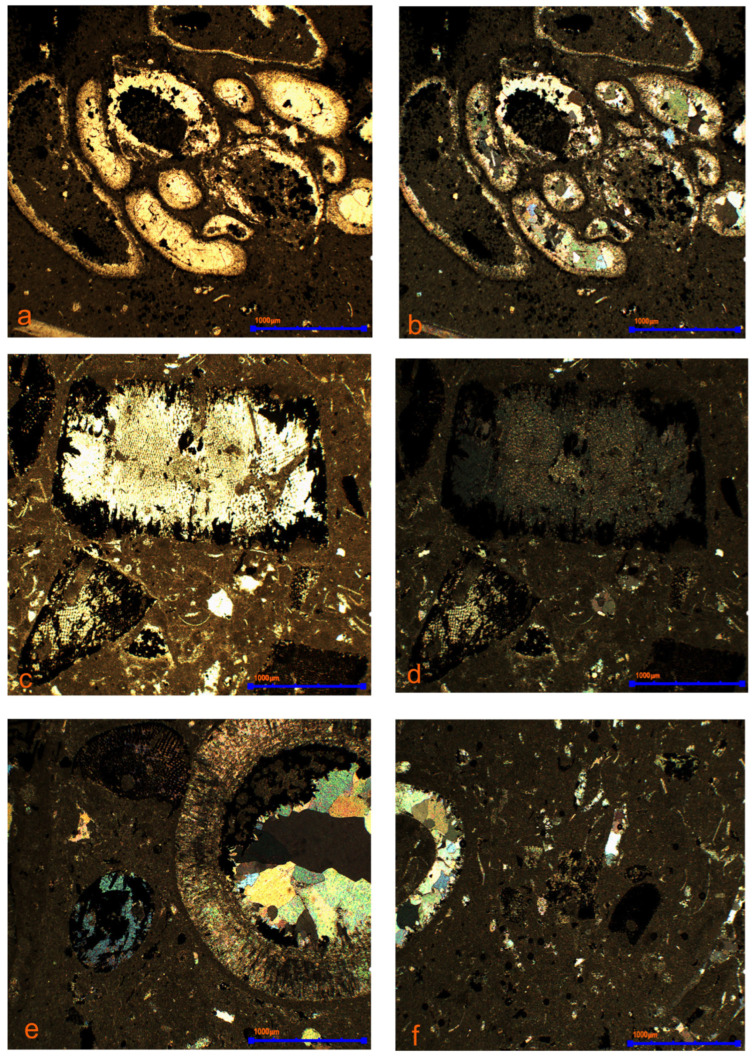
Organogenic limestone. Iron sulphides selectively fill organic remains. (**a**,**b**) carbonate sparite replaced by iron sulphides in ooids; (**c**,**d**) pyrite fills intracellular spaces in carbonate-type bioclasts. (**e**,**f**) selective replacement of carbonates with pyrite in bioclasts. Image of the polarising microscope 1P, 2P (explanations: P-polariser).

Pyrite replaces calcite in bioclasts, fills infaunal channels, stylolites, as well as fissures and cracks resulting from tectonic stresses, as well as pores and voids (Figure 11a,b). In organic remains, pyrite replaces the original skeleton material or fills its empty spaces. Carbonate sparite is replaced by iron sulphides in ooids (Figure 12a,b). Fragments of organisms are visible in the rocks where sulphide mineralisation occurred selectively (Figure 12c,d). Pyrite fills small spaces in the internal structures of the skeleton or often encrusts its surface in an uneven manner (Figure 12e,f). This situation occurs in partially metamorphosed organisms. The presence of unmetamorphosed organic remains was also found, as well as those where calcium carbonate was completely replaced by pyrite, precisely reflecting the original morphology of the skeleton. Pyritization in limestones is epigenetic in nature. The occurrence of various morphological forms of iron sulphides is the result of the presence of various types of organic remains, as well as the porosity of the skeleton [33]. The confirmed presence of framboidal forms is directly related to stylolite seams and infaunal channels.

The widespread occurrence of pyrite in the form of framboidal concretions proves that its crystallisation process was biochemical in nature and was preceded by the decomposition of sulphates caused by the activity of chemolithotrophic bacteria. These microorganisms use the oxygen contained in sulphides as an energy source, providing hydrogen sulphide, among others, for the crystallisation of sulphides. The formation of sharp-edged crystals from spherical forms is possible, provided that the supply of iron and sulphur is ensured during their growth [34,35]. Pyrite, which fills pores, voids, fissures, and tectonic cracks, is the result of crystallisation from ionic solutions.

## 4. Discussion

The results obtained from geochemical research indicate that the anomalous level of the determined elements in rock raw materials is of natural origin and is the result of the specific conditions of their formation. Their increased concentration is probably influenced by the Neogene lignite series and identified mineralisation processes. Lead and arsenic are associated with sulphide mineralisation. Cadmium is also associated with clay minerals.

Lead has sulphophilic tendencies, which is reflected in the formation of sulphide minerals. Organic remains and stylolite seams filled with iron sulphides and pyrite were observed in the raw materials tested. The mineralogical tests did not show any lead minerals in the samples, but pyrite accumulations. This sulphide is mentioned as a compound accompanying hydrothermal solution reactions with mainly carbonate rocks, in the galena formation process in the ore-bearing areas of dolomites from the Kraków-Silesia region [36].

The concentration of cadmium may be related to the weathering processes that the rocks in the zone studied were subjected to. Cadmium is an element strongly dispersed in rocks and is easily released during weathering processes. Then it is bound by clay minerals, iron hydroxides, and organic substances. This phenomenon is also influenced by the pyritization process identified in mineralogy studies. Some types of coal, peat, and crude oil contain relatively higher amounts of Cd, which may be due to its affinity for organic matter, selective adsorption, and complexation by humic compounds, especially sediments rich in organic substances and marine manganese and phosphite nodules [37]. It also appears that its increased concentration in Mesozoic basement rocks is due to the nature of lignite from the Bełchatów deposit, which is distinguished by a higher cadmium content compared to lignites observed around the world. It is assumed that its average concentration in the Bełchatów lignite is 0.6 mg/kg with a minimum value of <0.2 and a maximum of 0.6 mg/kg [38,39].

The determined arsenic content showed values that were elevated for the sandstones in which the pyritization process was also found. This element is associated with sulphide mineralisation and the influence of the lignite series. Arsenic-rich pyrite is common in sedimentary formations rich in organic matter, especially shale, lignite, and peat deposits [40,41,42]. Its concentration in lignite causes environmental problems and shows extreme variability among coals of different origins. It has been found to range from 0.5 to 80 mg/kg (average 10 mg/kg) [43], although higher values are sometimes reported. The As^5+^ form is adsorbed by sediments to a greater extent than other As ions [44,45]. Anthropogenic sources of arsenic include coal combustion, geothermal power plants, sulphide roasting, and ore smelting. Arsenic contamination of the environment as a result of mining and smelting is common [46,47]. Its presence in nature and from anthropogenic sources has perpetuated human exposure to this toxic and carcinogenic element [48]. Arsenic is released by the breakdown of pyrite and other sulphide minerals and can be incorporated by adsorption or other processes, causing changes in groundwater chemistry and increasing concentrations in the aquifer [49].

**Figure 13:**
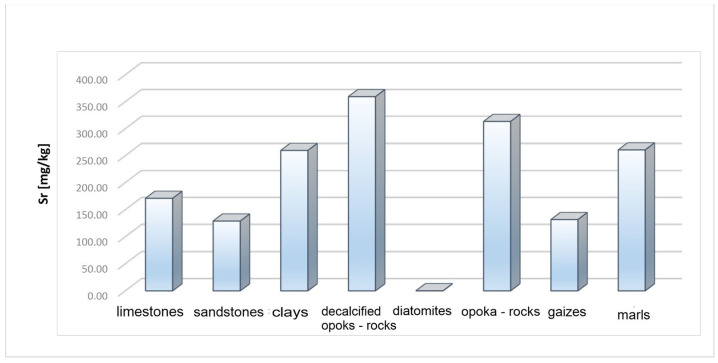
Average strontium content (Sr) in rock raw materials.

**Figure 14:**
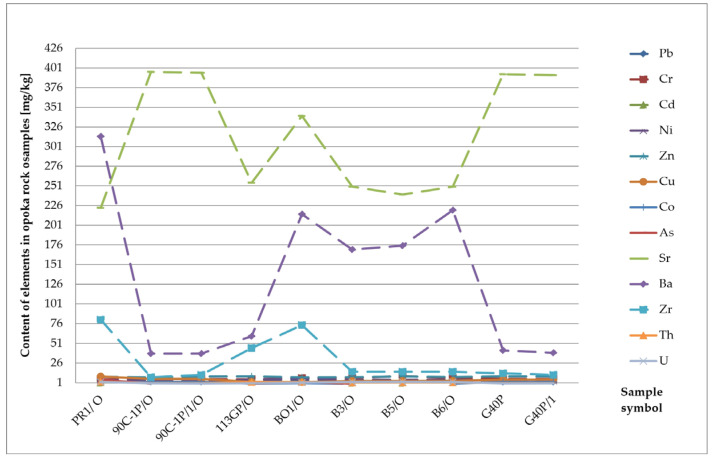
Distribution of strontium (Sr) in opoka rocks.

**Figure 15:**
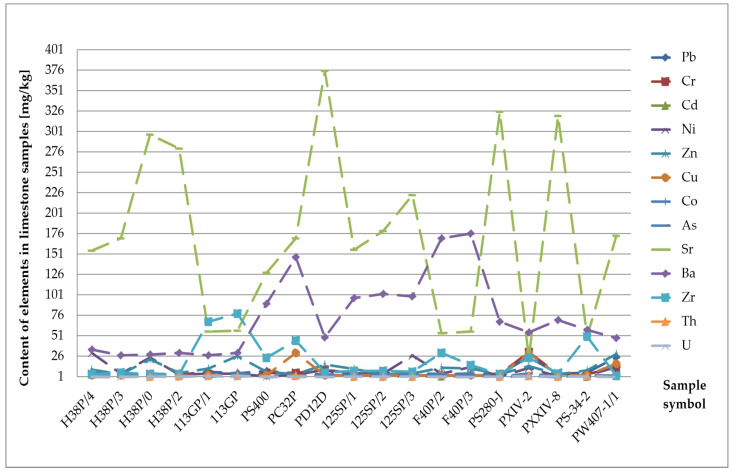
Distribution of strontium (Sr) in limestones.

**Figure 16:**
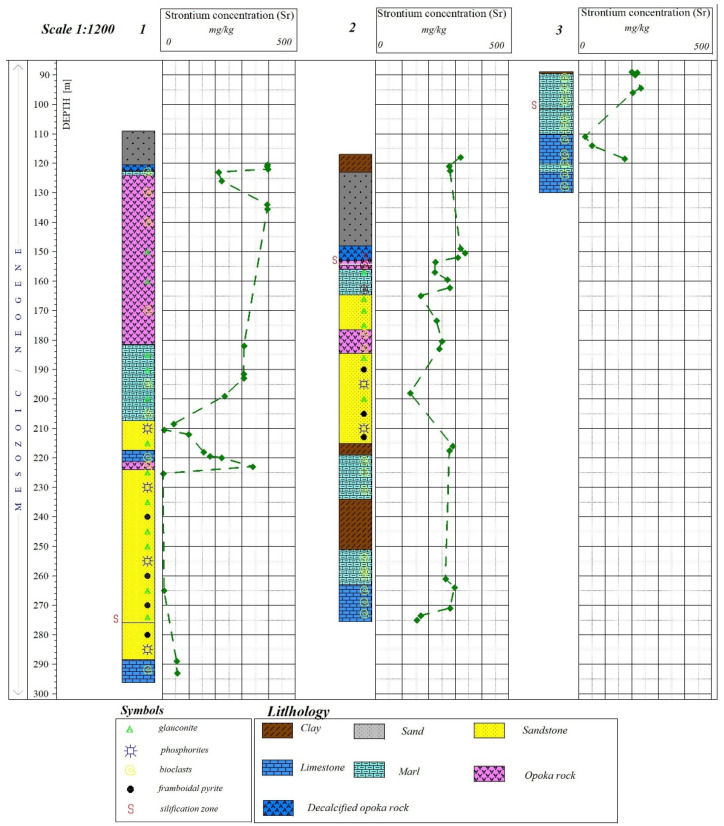
Examples of Sr distribution profiles in the tested rock raw materials.

High levels of strontium were recorded in all rocks. However, it can be observed that the highest mean strontium values were 359.34 mg/kg in decalcified opoka rocks and 313.4 mg/kg in opoka rocks (Figure 13). In these rocks, compared to limestones, strontium was recorded at a similar level in all samples. In limestones, the registered strontium had a wide range of values of 24–375 mg/kg with an average value of 171 mg/kg (Figure 14 and Figure 15, Table 1). The analysis of the profiles showed that the content of strontium (Sr) in these rocks decreases significantly at places where the silicification process has been identified. It should be noted that strontium increases from older geological carbonate formations (Jurassic limestones) to the younger Cretaceous rocks of Maastrichtian (Figure 16).

## 5. Conclusions

The results obtained from geochemical research indicate that the anomalous level of the determined elements (Cd, As, Pb) in the rock raw materials is of natural origin and is the result of the specific conditions of their formation. The geochemical nature of the rock raw materials studied is influenced by secondary mineralisation processes and the impact of the Neogene lignite series. The rocks examined were affected by two secondary processes: sulfatization and pyritization.

The sulfatization process has been identified in carbonate rocks. The occurrence of sulphate residues was found within some carbonate bioclasts. Celestine was identified in rock voids. These types of mineral phases can be associated with the activity of biochemical processes caused by the decomposition of the soft tissues of animal organisms and the organic substances of plant origin that fill the stylolites. The bacteriological reduction in sulphates led to the crystallisation of pyrite and granular sparite and ended with the beginning of the crystallisation of block sparite.

The pyritization process is epigenetic in nature. Pyrite selectively replaces calcite in bioclasts and fills infaunal channels, stylolites, and fissures and cracks resulting from tectonic stresses. The occurrence of various morphological forms of iron sulphides (from amorphous, framboidal, to isometric) is the result of the presence of various types of organic remains and the porosity of the skeleton.

Mineralised types of rock have higher concentrations of elements. The high values for cadmium (Cd), lead (Pb), and arsenic (As) are noteworthy. The cadmium content presented clearly indicates that it is overestimated relative to those considered as the limit level for the sedimentary rocks. Cadmium is an element strongly dispersed in rocks and is easily released during weathering processes. Then it is bound by clay minerals, iron hydroxides, and organic substances. It also seems that its increased concentration in Mesozoic basement rocks is due to the nature of the lignite from the Bełchatów deposit, which is distinguished by a higher cadmium content compared to lignites observed around the world. The increased Pb and As content was found only in the samples where the sulphide mineralisation process was recorded. The analysis of the obtained results draws attention to the increase in the concentration of strontium (Sr) with the age of the examined rocks and the weathering processes. On the basis of the results obtained, rock raw materials with the highest content of strontium (decalcified opoka rocks) were selected. The content of strontium increases from the carbonate formations of the geologically older Jurassic limestones to the younger Cretaceous rocks of Maastrichtian. The strontium content found in geochemical studies may be related to the weathering processes undergone by the rocks in the zone studied. During weathering processes, strontium dissolves, generally in the form of bicarbonate. It is easily sorbed by clay minerals and organisms that create limestone skeletons. Therefore, not only are carbonate minerals responsible for the accumulation of strontium but also the processes to which the given rocks were subjected. This is confirmed by the higher content of this element in the decalcified opoka rocks than in the opoka rocks or marls. This information can be important for monitoring the source of the materials. Moreover, the detected content of strontium may have economic and economic significance, and this element is listed among the critical and strategic raw materials of the European Union. The geochemical tests carried out indicate that the remaining elements determined in the tested rock raw materials do not exceed the permissible content.

## Figures and Tables

**Table 1:** Content of elements in the rock raw materials tested [mg/kg].

Content of Element Min–Max (Mean) [mk/g]	Lithological Type of Rocks							
	Limestones	Sandstones	Kaolin Clays	Decalcyfied Opoka Rocks	Opoka Rocks	Diatomites	Marls	Gaizes
Pb	3.44–24.94 (7.01)	2–24.94 (9.82)	12–30 (20.1)	0	2.2–3.3 (2.76)	0–4.3 (2.29)	1.72–8.06 (4.54)	2.5–4.3 (3.24)
Cr	0–30.82 (7.57)	1.43–56 (15.82)	82–99 (93)	0–0.05 (0.04)	5.36–9.40 (6.52)	0.72–18.76 (8.37)	0–9.3 (5.9)	0.02–5.36 (3.13)
Cd	0–5.28 (1395)	0–1.54 (0.86)	0.3–0.5 (0.42)	2.01–2.64 (2.13)	0–1.1 (0.55)	0–0.44 (0.44)	0–1.1 (0.89)	0–0.22 (0.21)
Ni	1.1–30 (8.86)	1–20 (8.99)	42–60 (50.2)	0	3.25–6 (4.54)	0–18.56 (6.13)	0–3.48 (2.80)	2.22–8.12 (4.41)
Zn	3.79–28.75 (10.81)	2–87 (26.88)	66–113 (95)	1.2–1.58 (1.33)	7.99–9.05 (8.63)	4.58–77.1 (24.79)	4.26–46.29 (19.44)	3–13.43 (6.97)
Cu	0–30 (6.6)	1–92 (17.21)	26–57 (47.6)	1.5–2.8(2.35)	1.9–9 (4.53)	2.8–11.2 (6.74)	2.8–30 (5.76)	1.95–22.4 (10.29)
Co	0.1–6 (2.76)	0.1–7 (2.81)	9–14 (10.4)	0–001(0.01)	0–2.21 (1.6)	1–4 (1.9)	0–6 (2.92)	0–4 (3.7)
As	0–5 (1.84)	1–5 (2.67)	2–4 (2.83)	0	0–5.1 (2.44)	0.05–9.6 (6.83)	0–2 (2)	1.5–4.3 (2.59)
Sr	24–375 (170.94)	3–325 (128.92)	200–320 (259.6)	320–398 (359.34)	223–396 (313.4)	0–0.05 (0.05)	203–308 (260.6)	49–222 (131.6)
Ba	27–176 (73.94)	23–145 (56.14)	219–340 (273.1)	37–40 (38.66)	38–314 (131.1)	185–210 (196)	25–158 (60.1)	120–190 (144)
Zr	2–78 (20.63)	2–698 (166)	120–193 (168.7)	7–10 (8)	8–81 (28.8)	18.5–20 (19.1)	2–36 (19.2)	17.5–100 (51.9)

**Table 2:** Mineral composition of rock raw materials.

Types of Rocks	Mineral Composition
Limenstones	Calcite Quartz, Feldspar, Pyrite, Apatite, Celestine, Barite, Gypsum, Siderite, Kaolinite
Sandstones	Quartz Feldspars, Micas, Glauconite, Pyrite, Zircon, Heavy minerals
Clays	Kaolinite Illite, Feldspar, Quartz
Decalcified opoka-rocks	Chalcedony Kaolinite, Quartz, Calcite, Zircon, Rutile, Tourmaline, Pyrite
Opoka-rocks	Calcite, Opal-CT Quartz, Chalcedony, Monazite
Diatomites	Opal-CT, Quartz Glauconite, Feldspars, Pyrite, Zeolites, Montmorillonite, Illite, Muscovite
Marls	Calcite, Opal, Smectite minerals Quartz, Glauconite, Biotite, Muscovite, Celestine, Pyrite
Gaizes	Chalcedony, Opal-CT Illit

## Data Availability

Data are contained within the article.
